# Measurement of fractionated plasma metanephrines for exclusion of pheochromocytoma: Can specificity be improved by adjustment for age?

**DOI:** 10.1186/1472-6823-5-1

**Published:** 2005-02-28

**Authors:** Anna M Sawka, Lehana Thabane, Amiram Gafni, Mitchell Levine, William F Young

**Affiliations:** 1 Department of Internal Medicine, St. Joseph's Healthcare, Hamilton, Ontario, L8N 4A6, Canada; 2 Division of Endocrinology and Metabolism, McMaster University, Hamilton, Ontario, L8N 3Z5, Canada; 3 Centre for Evaluation of Medicines, St. Joseph's Healthcare, Hamilton, Ontario, L8N 1G6, Canada; 4 Department of Clinical Epidemiology and Biostatistics, McMaster University, Hamilton, Ontario, L8N 3Z5, Canada; 5 Division of Endocrinology, Metabolism, Nutrition, and Internal Medicine, Mayo Clinic, Rochester, MN, 55905, USA

## Abstract

**Background:**

Biochemical testing for pheochromocytoma by measurement of fractionated plasma metanephrines is limited by false positive rates of up to 18% in people without known genetic predisposition to the disease. The plasma normetanephrine fraction is responsible for most false positives and plasma normetanephrine increases with age. The objective of this study was to determine if we could improve the specificity of fractionated plasma measurements, by statistically adjusting for age.

**Methods:**

An age-adjusted metanephrine score was derived using logistic regression from 343 subjects (including 33 people with pheochromocytoma) who underwent fractionated plasma metanephrine measurements as part of investigations for suspected pheochromocytoma at Mayo Clinic Rochester (derivation set). The performance of the age-adjusted score was validated in a dataset of 158 subjects (including patients 23 with pheochromocytoma) that underwent measurements of fractionated plasma metanephrines at Mayo Clinic the following year (validation dataset). None of the participants in the validation dataset had known genetic predisposition to pheochromocytoma.

**Results:**

The sensitivity of the age-adjusted metanephrine score was the same as that of traditional interpretation of fractionated plasma metanephrine measurements, yielding a sensitivity of 100% (23/23, 95% confidence interval [CI] 85.7%, 100%). However, the false positive rate with traditional interpretation of fractionated plasma metanephrine measurements was 16.3% (22/135, 95% CI, 11.0%, 23.4%) and that of the age-adjusted score was significantly lower at 3.0% (4/135, 95% CI, 1.2%, 7.4%) (p < 0.001 using McNemar's test).

**Conclusion:**

An adjustment for age in the interpretation of results of fractionated plasma metanephrines may significantly decrease false positives when using this test to exclude sporadic pheochromocytoma. Such improvements in false positive rate may result in savings of expenditures related to confirmatory imaging.

## Background

Pheochromocytoma is a rare tumor of the adrenal medulla or sympathetic ganglia, which can secrete excessive catecholamines [1]. Signs and symptoms of pheochromocytoma may include hypertension, pain (including headache, flank pain, abdominal pain, or chest pain), hyperhidrosis, anxiety or panic attacks, cardiac arrythmias, or sudden death [1-6]. A pheochromocytoma may also be detected as an asymptomatic incidental adrenal mass seen on abdominal imaging [7]. Metabolites of norepinephrine and epinephrine, specifically normetanephrine and metanephrine, may be measured in the plasma by high performance liquid chromatography with electrochemical detection, as described by Lenders et al. [8]. Measurement of fractionated plasma metanephrines has been called "the best test for excluding or confirming pheochromocytoma" and some investigators have recommended that such measurements "should be the test of first choice" [9]. In a recent systematic review of the world literature, we have observed that measurement of fractionated plasma metanephrine measurements have a high sensitivity ranging of 96 to100% and a variable specificity ranging from 82% to 100% [10]. The specificity of fractionated plasma metanephrines in excluding pheochromocytoma appears lowest in populations without known genetic predisposition to disease (those in whom sporadic disease is sought), with a false positive rate of up to 18% in such patients [9]. We have previously observed that the normetanephrine fraction is elevated in the majority of false positive test results and that false positives are associated with increasing age [11]. Indeed, investigators from the National Institute of Health have agreed that, "measurements of plasma normetanephrine and metanephrine provide a highly sensitive test for diagnosis of pheochromocytoma, but false positive results remain a problem" [12]. False positive biochemical test results may result in needless imaging procedures and generate excessive healthcare expenditures in detection of sporadic pheochromocytoma [13].

Reasons for false positive fractionated metanephrine test results have been explored and alternatives for further evaluation of patients with positive test results have been proposed. It is known that acetaminophen may interfere with measurements of fractionated plasma metanephrines using the Lenders' method [8], so this drug has traditionally been avoided prior to testing. Eisenhofer and colleagues have also suggested that that tricyclic antidepressants and phenoxybenzamine, respectively, may result in false positive tests [12]. Of note, in a recent Mayo study, 13% of subjects with false positive fractionated metanephrines used tricyclic antidepressants [11] but 9% of subjects who had normal fractionated metanephrine measurements also used these drugs. Thus, tricyclic use did not seem to explain the majority of false positives seen at the Mayo Clinic. Furthermore, given that phenoxybenzamine is rarely used in patients without known pheochromocytoma, use of this drug does not explain why there are so many false positive fractionated metanephrine results observed in clinical practice. As a method to distinguish false positives from true positives, Eisenhofer and colleagues have recommended clonidine-suppression testing in patients with positive fractionated plasma metanephrine measurements [12]. Eisenhofer and colleagues have recommended that plasma norepinephrine and normetanephrine levels be measured three hours after a dose of 0.3 mg clonidine in such patients [12]. We have proposed an alternative strategy to deal with false positive test results in patients without known genetic predisposition to disease [13]. As normetanephrine is the fraction responsible for the majority of false positive results, we have proposed that 24-hour urinary measurements of fractionated catecholamines and metanephrines be performed in patients with normetanephrine elevations that are approximately one and two times the upper limit of the normal range, to confirm the biochemical presence of sporadic pheochromocytoma [13]. Of note, in the case of high risk patients with known genetic predisposition to pheochromocytoma, the pre-test probability of disease may be sufficiently high that measurement of fractionated plasma metanephrines, without confirmatory biochemical studies may be reasonable [11]. Thus, the issue of lack of specificity of fractionated plasma metanephrine measurements is applicable primarily to non-genetically predisposed individuals in whom sporadic pheochromocytoma is sought.

Our objective was to determine whether an adjustment of fractionated plasma metanephrines for age may improve the specificity of interpretation of these measurements when biochemically excluding sporadic pheochromocytoma, and thus result in costs savings in confirmatory imaging studies.

## Methods

### Study populations

The age-adjusted fractionated plasma metanephrine score was derived from a previously described dataset of 349 subjects (including 33 people with pheochromocytoma) who underwent measurement of fractionated plasma metanephrines as part of an evaluation of suspected pheochromocytoma (also known as the derivation set) [11]. The diagnostic efficacy of this logistic-regression derived prediction rule was then tested in a second dataset of 158 subjects (including 23 with sporadic pheochromocytoma) who had measurements of fractionated plasma metanephrines performed at the Mayo Clinic Rochester the following year (the validation dataset). None of the patients in the validation set had known genetic predisposition to pheochromocytoma. All patients with pheochromocytoma had histologic confirmation of the diagnosis and those without pheochromocytoma had an alternative diagnosis assigned at the completion of their evaluation based on a combination of other biochemical test results (such as normal 24-hour urinary fractionated metanephrine and catecholamine measurements with or without normal imaging of the adrenals in the form of computerized tomography scanning [CT] or magnetic resonance imaging [MRI]). All data were obtained by retrospective chart review. The Institutional Review Board at Mayo Clinic Rochester approved the study.

### Measurement of fractionated plasma metanephrines

The technique of Lenders (by high performance chromatography and electrochemical detection) was used to measure fractionated plasma metanephrines [8]. The traditional criterion for positivity is a metanephrine fraction greater than or equal to 0.5 nmol/L or a normetanephrine fraction greater than or equal to 0.9 nmol/L, based on a 95% reference range derived by Mayo Medical Laboratories. Subjects were advised to avoid acetaminophen for 48 hours prior to measurement of fractionated plasma metanephrines. Fractionated plasma metanephrine measurements were taken in the sitting position, with no indwelling venous cannula, and no dietary restrictions prior to testing. The lower limit of detection for the normetanephrine and metanephrine fractions was 0.20 nmol/L. Therefore, normetanephrine and metanephrine fractional measurements reported as being below the detection limit were given the value of 0.19 nmol/L for use in the logistic regression formula. Subjects who had "interfering substances" reported by the laboratory on measurement of fractionated plasma metanephrines were excluded from analyses but the number of such subjects was recorded.

### Statistical methods

Clinical characteristics of subjects in the derivation set who did not have pheochromocytoma but had measurements of the normetanephrine or metanephrine fraction above the upper reference limits (false positive tests using traditional positivity criteria) were compared to those without pheochromocytoma who had true negative tests (χ^2 ^was used for categorical variables and Student's t-test for independent samples was used for continuous variables). Variables which were different between both groups at a significance level of 0.1 were then entered into a multivariable logistic regression model predicting pheochromocytoma. Age was the only variable of statistical significance distinguishing true positive from false positive fractionated plasma metanephrine measurements in the derivation set. Thus, we forced age with measurement values of normetanephrine and metanephrine fractions in a multivariable logistic regression model predicting pheochromocytoma in the derivation set (SPSS 10.0, Chicago, ILL). The formula for this age-adjusted fractionated plasma metanephrine score is shown below:

*-4.188 + -0.07(age) + 4.516(metanephrine) + 3.129(normetanephrine)*.

Age was in years, metanephrine fraction in nmol/L and normetanephrine fraction in nmol/L in this formula. In the derivation set, the Hosmer and Lemeshow test had χ^2 ^= 4.73, df = 8, p = 0.79 and the Cox and Snell r^2 ^= 0.38 showing significant model goodness-of-fit. A positivity cut-off age-adjusted metanephrine value of = -1.4752 was chosen as it carried an acceptable sensitivity of = 90.9% (30/33 patients, 95% CI = 76.4%, 96.9%) and specificity of 96.8% (300/310 patients, 95% CI = 94.2%, 98.2%) in the derivation set. The sensitivity level of over 90% was chosen because such a sensitivity level was believed to be clinically reasonable and at this level, specificity was still acceptable. We were aware that the lower the cut-off, the higher the sensitivity, but this would be at the expense of specificity.

The formula for adjustment of age was applied to fractionated plasma metanephrine measurements in the second dataset (validation dataset) for testing of sensitivity and specificity in a population in whom sporadic pheochromocytoma was sought. In the validation set, individuals with known genetic predisposition to pheochromocytoma were excluded. Sensitivities were calculated by division of subjects with true positive test results by all the subjects with pheochromocytoma, and specificities were calculated by division of subjects with true negative test results divided by all subjects without pheochromocytoma. For sensitivities, specificities, and likelihood ratios, 95 percent confidence intervals (CI) were calculated using Wilson's method (except the Score Method was used for calculation of 95% CI of likelihood ratios when a zero cell was noted) [14]. The specificity of the age-adjusted metanephrine score were compared (at the same level of sensitivity) to traditional interpretation of fractionated plasma metanephrine measurements using McNemar's test [15].

### Economic evaluation (decision analysis)

We investigated whether use of an age-adjusted fractionated plasma metanephrine measurement could result in cost savings in imaging expenditures, compared to use of fractionated plasma metanephrine measurements interpreted in a conventional fashion, for detection of sporadic pheochromocytoma in a hypothetical tertiary care hypertensive population. We thus performed a decision analysis, with resource implications defined by costs of confirmatory imaging (CT and MRI), interpreted from a third party payer perspective. In the decision analysis, we compared algorithm "A" in which biochemical testing consisted of measurement of fractionated plasma metanephrines with these measurements interpreted relative to the 95% reference range (defined by a normetanephrine fraction above 0.9 nmol/L or a metanephrine fraction above 0.5 nmol/L) to algorithm "B", in which fractionated plasma metanephrine measurements were interpreted by using the age-adjusted metanephrine score. The sensitivity and specificity of biochemical tests was based on data from the validation set. In each algorithm, all patients with positive biochemical testing would undergo confirmatory imaging. The imaging protocol for patients with positive biochemical tests in either strategies began with computerized tomography ([CT] with and without intravenous contrast) of the abdomen, then if negative, I-131 or I-123 metaiodobenzylguanidine (MIBG) scintigraphy (efficacy for I-131 and costs for I-123 shown). The horizon (endpoint) of the analyses was positive diagnosis or exclusion of pheochromocytoma, for hypothetical hypertensive patients subjected to each strategy. The outcome of the analyses was the number of patients with pheochromocytoma expected to be detected by each strategy. The costs of false positive biochemical tests were reflected only in the costs of subsequent imaging and not in potential costs of needless surgery or its possible complications.

The diagnostic efficacy of imaging studies was based on respective estimates from the literature: The sensitivity of CT imaging of the abdomen was assumed to be 98% with a specificity of 70% [16]. The sensitivity of MIBG in detecting benign sporadic pheochromocytoma was assumed to be 87.4% with a specificity of 98.9% [17].

All costs were reported in 2002 US dollars. Costs of imaging investigations were obtained from the Mayo Clinic Rochester Business Office. For the purpose of the decision analysis model, the prevalence of pheochromocytoma in the hypertensive population that would typically be screened was assumed to be 0.5% [18].

## Results

### Findings in the derivation set

The derivation set (from which the age-adjusted score was developed) consisted of 349 consecutive subjects (including 33 people with pheochromocytoma) who underwent fractionated plasma metanephrine measurements as well as 24-hour urinary total metanephrine measurements with or without 24-hour urinary catecholamine measurements in testing for pheochromocytoma at Mayo Clinic Rochester. In the derivation set, 8 of the 33 individuals had clinically-diagnosed genetic syndromes predisposing to pheochromocytoma (three familial malignant paraganglioma, two von Hippel-Lindau, one had multiple endocrine neoplasia 2a, one had multiple endocrine neoplasia 2b, and one had familial multiple benign paraganglioma). The 316 individuals in the derivation set who did not have pheochromocytoma underwent such testing the following reasons: refractory hypertension (174, 55%), spells (periodic episodes of symptoms such as palpitations, headache, or sweating, 124, 39%), adrenal mass (45, 14%), previous pheochromocytoma or known genetic predisposition to pheochromocytoma (24, 8%). The mean age of subjects with pheochromocytoma was 48 years (SD 18 years, range 16 to 60 years), whereas the mean age of subjects without pheochromocytoma was 52 years (SD 15 years, range 10 to 73 years). Six of the 316 subjects without pheochromocytoma in the derivation set did not have a plasma metanephrine fraction recorded secondary to "interfering substances" and therefore were excluded from the analyses. In the derivation set, the sensitivity of traditionally interpreted fractionated plasma metanephrine measurements (using 95% reference ranges) was 93.9% (95% CI, 80.4, 98.3) (31/33 subjects), with a specificity of 85.2% (95% CI, 80.8, 88.7) (264/310 subjects). Baseline characteristics of individuals in the derivation set without pheochromocytoma were compared for individuals who had true negative fractionated metanephrine measurements (n = 264) to those who had false positive results (n = 46) (Table 1). The individuals with false positive fractionated plasma metanephrine measurements in the derivation set were significantly older than those with true negative measurements (p = 0.007), whereas blood pressure, antihypertensive medication use, and rates of obstructive sleep apnea were not significantly different between these groups. Thus, age was chosen as an important variable to adjust for in interpretation of fractionated plasma metanephrines and an age-adjusted metanephrine score was developed from the derivation set data using logistic regression (as described in the Methods). At a cut-off value of -1.4752, the sensitivity of the age-adjusted metanephrine score was 90.9% (30/33 patients, 95% CI, 76.4%, 96.9%), with a specificity of 96.8% (300/310 patients, 95% CI, 94.2%, 98.2%). In this derivation set, which included individuals genetically predisposed to pheochromocytoma, one individual with a dopamine-secreting paraganglioma, another patient with a von Hippel-Lindau disease (diagnosed clinically), and a third patient with sporadic pheochromocytoma had false-negative age-adjusted metanephrine scores. The efficacy of the age-adjusted metanephrine score was then validated in the validation set.

**Table 1:** Clinical characteristics of subjects without pheochromocytoma from the derivation set

**Clinical characteristic**	**True negative Measurements of fractionated plasma metanephrines (n = 264)**	**False positive Measurements of fractionated plasma metanephrines (n = 46)**	**Significance testing results**
**Gender (females/n)**	142/264	26/46	P = 0.73 (χ^2 ^= 0.12, df = 1)
**Age (mean, SD years)**	50.7 (15.03)	57.3 (14.6)	P = 0.007 (t = 2.81, df = 63)
**Systolic blood pressure (mean, SD mmHg)**	147 (26) (n = 260)	153 (32)	P = 0.24 (t = 1.19, df = 56)
**Diastolic blood pressure (mean, SD mmHg)**	87 (12) (n = 260)	89 (15)	P = 0.39 (t = 0.87, df = 57)
**Number of antihypertensive agents currently used (mean, SD)**	1.4 (1.5)	1.6 (1.3)	P = 0.28 (t = 1.09, df = 70)
**Known diagnosis of obstructive sleep apnea**	15/264	4/46	P = 0.43 (χ^2 ^= 0.62, df = 1)

### Findings in the validation set

In the validation set of 158 subjects, 23 patients had histologically-proven sporadic pheochromocytoma (17 adrenal, 6 extra-adrenal, 8 malignant). Of the patients with pheochromocytoma, none were known to be genetically predisposed to pheochromocytoma and 14/23 were women (61%). The mean age of subjects with pheochromocytoma was 50 years (SD 16 years, range 16 to 83 years), whereas the mean age of subjects without pheochromocytoma was 55 years (SD 16 years, range 7 to 86 years). Of the 135 subjects without pheochromocytoma, 83 (62%) were women. Reasons for measurement of fractionated plasma metanephrines in the subjects without pheochromocytoma were as follows: hypertension (55, 41%), spells with or without hypertension (44, 33%), an incidentally discovered adrenal mass (20, 15%), and previously surgically cured pheochromocytoma (16, 12%).

**Figure 1:**
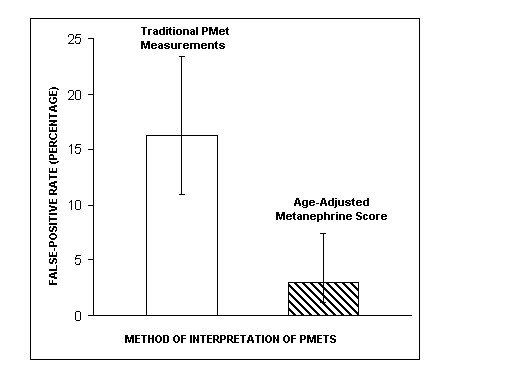
**Percentage of false positive test results (and 95% confidence interval) at 100% sensitivity in using a traditional interpretation of fractionated plasma metanephrine measurements or an age-adjusted metanephrine score. **Legend – Pmet(s), fractionated plasma metanephrine measurements; traditional fractionated plasma metanephrine measurements are considered positive if the metanephrine fraction is greater than or equal to 0.5 nmol/L or the normetanephrine fraction is greater than or equal to 0.9 nmol/L; an age-adjusted metanephrine score is positive if it is greater than -1.4752. The difference between false positive rates is statistically significant with p < 0.001 using McNemar's test.

In the validation set, the sensitivity of the age-adjusted metanephrine score was the same as the traditional interpretation of fractionated plasma metanephrine measurements at 100% (23/23, 95% CI, 85.7%, 100%). The specificity of the traditional interpretation of fractionated plasma metanephrine measurements was 83.7% (113/135 patients, 95% CI, 76.6%, 89.0%) and the specificity of the age-adjusted plasma metanephrine score was 97.0% (131/135 patients, 95% CI, 92.6%, 98.8%). Thus, the false positive rate with traditional interpretation of fractionated plasma metanephrine measurements was 16.3% (22/135, 95% CI, 11.0%, 23.4%) and with the age-adjusted score it was significantly lower at 3.0% (4/135, 95% CI, 1.2%, 7.4%) (Figure 1) (p < 0.001 using McNemar's test).

### Imaging cost implications of screening strategies for pheochromocytoma

**Figure 2:**
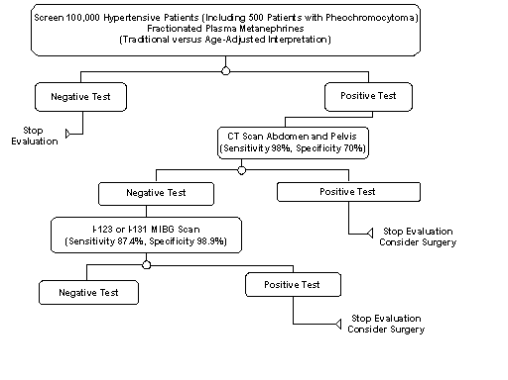
Decision analysis: Testing algorithm for pheochromocytoma in 100,000 hypothetical hypertensive subjects (including 500 individuals with pheochromocytoma)

In the decision analysis, biochemical testing by measurement of fractionated plasma metanephrines (traditional versus age-adjusted interpretation) was followed by CT imaging for all positive biochemical tests and if CT imaging was negative, then MIBG (I-123 or I-131) would be performed (Figure 2). For the purpose of the economic evaluation, in all three screening strategies, a 0.5% prevalence of pheochromocytoma was assumed in a target hypertensive population, so 500 patients with pheochromocytoma would be expected in a sample of 100,000 hypertensive subjects (Figure 2). Mayo Clinic Rochester charges for diagnostic studies were used: CT scan of the abdomen (with and without contrast) $1460, I-123 MIBG scan (with and without spect) $1875.

If 100,000 subjects with hypertension would be screened using algorithm "A" (beginning with biochemical testing by measurement of fractionated plasma metanephrines, traditional interpretation), 499/500 patients with pheochromocytoma (overall sensitivity 99.8%) would be expected to be detected (1 patient expected to have false negative CT and MIBG imaging); furthermore 94,510/99,500 of subjects without pheochromocytoma would be reassured with a negative diagnosis (overall specificity 95.0%). In algorithm "A", 16,718 individuals would undergo CT scanning and 11,363 individuals would undergo I-123 or I-131 MIBG imaging. The total cost of imaging for algorithm "A" would be estimated to be 45.7 million dollars.

If algorithm "B" (biochemical testing using the age-adjusted metanephrine score) would be used in 100,000 subjects with hypertension, 499/500 patients with pheochromocytoma (99.8%) would be expected to be detected and 98592/99,500 individuals without pheochromocytoma would be reassured with a negative test result (overall specificity of 99.1%). In algorithm "B", 3,485 individuals would undergo CT scanning and 2,100 individuals would undergo I-123 or I-131 MIBG imaging. The cost of imaging for algorithm "B" would be approximately 9.0 million dollars. Thus, use of the age-adjusted plasma metanephrine score for biochemical testing for sporadic pheochromocytoma in a hypothetical population of 100,000 tertiary care hypertensive patients could result in a cost savings of 36.7 million dollars with equal detection of pheochromocytoma cases, relative to using the same biochemical testing but interpreting fractionated plasma metanephrine measurements in a traditional fashion.

## Discussion

We agree with observation by Eisenhofer and colleagues that when it comes to measurement of fractionated plasma metanephrines for exclusion of pheochromocytoma, "false-positive results remain a problem" [12], particularly when attempting to exclude sporadic disease. Originally, it was the hope was that measurement of fractionated plasma metanephrines could result in cost savings because of avoidance of multiple biochemical tests [19]. However, investigators from the National Institute of Health have recommended that clonidine-suppression tests need to be done in order to distinguish true positives from false positives [12]. An alternative to clonidine-suppression testing may be measurement of 24-hour urinary metanephrines and catecholamines in patients with mild to moderate elevations of the normetanephrine fraction (for normetanephrine values approximately one to two times the upper limit of the normal range) [13]. In our study, we have provided a unique alternative approach for improving specificity of interpretation of measurements of fractionated plasma metanephrines. By adjusting the metanephrine score for age, we have shown that it may be possible to improve specificity of interpretation of fractionated plasma metanephrines, with no loss of sensitivity in detection of sporadic pheochromocytoma, and potential savings in imaging expenditures.

Of note, the sensitivity of the age-adjusted metanephrine score was superior in the validation set (100%) to that observed in the original dataset from which it was derived (91%). An explanation for this finding may be that the validation set included only people who were at risk for sporadic pheochromocytoma (in other words, non-genetically predisposed individuals), whereas genetically predisposed individuals were included in the original derivation dataset. We have previously observed that fractionated plasma metanephrine measurements may be normal in genetically-predisposed individuals with small pheochromocytomas [11]. Moreover, the physiologic cause for the observed relationship of normetanephrine measurements with age is unclear. Of note, Raber et al have noted exaggerated increases in plasma normetanephrine after exercise in hypertensive individuals with type 2 diabetes, compared to normotensive individuals with or without diabetes [20]. Furthermore, Raber et al have suggested that the excessive response of plasma normetanephrine to exercise may serve as a marker of exaggerated sympathoadrenal function in hypertensive type 2 diabetics [20]. Fractionated plasma metanephrine measurements were performed only at rest in our study and we did not examine any potential relationship with diabetes. Systolic and diastolic blood pressures were not significantly different between individuals with false positive fractionated metanephrine measurements and those with true negative measurements in the derivation set in our study.

Our study is subject to several limitations. Firstly, limited clinical data on each studied individual were collected so variables that could be of interest such as: body mass index, creatinine-clearance, and rates of diabetes mellitus were not recorded. Furthermore, without autopsy confirmation, one cannot be absolutely certain that individuals labelled as not having a pheochromocytoma did not have an occult paraganglioma or pheochromocytoma. However, we believe that reasonable clinical criteria were used in excluding pheochromocytoma in our study. Another limitation is that we used an assay for measurement of fractionated plasma metanephrines that may be have been subject to interference with acetaminophen [8], whereas other assays, such as the one described by Roden et al, could have been preferable due to lack of acetaminophen interference [21]. The cut-off that we chose for positivity of the age-adjusted metanephrine score was also arbitrary and use of a lower cut-off could have resulted in improved sensitivity, albeit with likely some expense of specificity. Finally, our findings have not been validated outside a single institution.

Is calculation of an age-adjusted metanephrine score practical for use in daily clinical practice? In this day of palm pilots and desktop computers, we believe that it may be feasible for clinicians to enter the formula for age-adjustment into standard desktop spreadsheet software (such as Excel, Microsoft) and perform such adjustments in the clinic. Alternatively, laboratories can provide age adjusted values to physicians when reporting test results. Thus, we do believe that calculation of an age-adjusted score is feasible to assist physicians in interpretation of fractionated plasma metanephrine measurements. Indeed, such calculations may be less cumbersome and may generate fewer healthcare expenditures than alternative procedures such as supplemental clonidine-suppression testing or collection of 24-hour urinary measures.

Our observations should, however, be validated in another population outside of Mayo Clinic. Of particular note, our findings are applicable only to the screening of pheochromocytoma in low risk subjects who do not have known genetic predisposition to disease. In high risk, genetically predisposed individuals, mild elevations of normetanephrine or metanephrine measurements may be clinically significant and should prompt imaging.

## Conclusion

An adjustment for age in interpretation of results of fractionated plasma metanephrine measurements may significantly improve the high false positive rate seen with this test when aiming to exclude sporadic pheochromocytoma. This improvement in specificity may result in savings in expenditures related to confirmatory imaging. Additional research is needed to investigate the generalizability of these findings in other clinical centres.

## Competing interests

Dr. Sawka is a Skeletal Health Scholar funded, in part, by the Canadian Institutes of Health Research. Dr. Sawka was also a Fellow in Health Economics at McMaster University, partly funded by an unrestricted educational grant from Hoffmann-La Roche.

The other co-authors have no competing interests to declare.

## Authors' contributions

All co-authors reviewed the manuscript and made suggestions for revisions. The project idea was conceived by A.M. Sawka. Analyses were performed by A.M. Sawka, with input from Dr. Thabane. The manuscript was written and revised by Dr. Sawka.

## Pre-publication history

The pre-publication history for this paper can be accessed here:


